# Supplementary material for: Novel Harmonization Method for Multi-Centric Radiomic Studies in Non-Small Cell Lung Cancer
Source: Curr Oncol. 2022 Jul 22;29(8):5179–94. doi: 10.3390/curroncol29080410 (PMC9332210; doi:10.3390/curroncol29080410)
Supplement: Supplementary file 1 [file curroncol-29-00410-s001.zip › curroncol-1731966-supplementary.pdf]

Supplementary material

Table S1. - The image preprocessing of intensity and spatial discretization.

Pre-processing settings	Spatial resampling	Re-segmented range	Bin width	Gray levels
CT	$[1.0 \times 1.0 \times 1.0] mm^3$	$[-1000 \div 3000]\ HU$	10 HU	400
PET	$[3.0 \times 3.0 \times 3.0] mm^3$	[0 ÷ 20] SUV	0.3125 SUV	64

Table S2. - Lasso feature selection coefficients.

Models' condition	λ_{LASSO}
Harmo CT + Original PET features (A)	0.1355
Harmo CT features (B)	0.1220
Original CT features (C)	0.1290
PET features only (D)	0.0950

Text S1. Models' description

Linear SVM

Linear SVM classifiers provide low generalization error even with small learning sample datasets. A binary classification problem can be viewed as the task of separating observations in the features space. Consider a training dataset defined through a matrix$n \times p$, and that these observations fall in two different classes collected in a vector of dimension$n$. The aim is to classify unseen observations belonging to the validation dataset.

The basic idea under the SVM classifier is to find an optimal hyperplane that separates the observations in the features space. One can easily determine on which side of the hyperplane a point lies, by simply calculating the sign of the hyperplane mathematical expression. Also, it is possible to compute the perpendicular distance between each observation and the hyperplane. The smallest distance between the observations and the hyperplane is called margin. In real classification problems, the hyperplane cannot separate perfectly the data into two classes. Because it isn't possible to define exactly the side on which the observations are located the SVM classifier allows the observation to be on the wrong side of the hyperplane. Setting the hyperplane equation in p dimension as $\beta_0 + \beta_1 X_1 + \beta_2 X_2 + \cdots + \beta_p X_p = 0$, where X_i are the observations and β_i the parameters, and M as the margin, the optimal classifier can be found by solving the quadratic optimization problem that maximizes the separation margin between the closest data points of each class, referred to as the support vectors:

$$\begin{cases} Maximize_{\beta_0,\ldots,\beta_p,\epsilon_1,\ldots,\epsilon_n} M \\ Subjected\ to \sum_{j=1}^{n} \beta_j = 1 \\ y_i(\beta_0 + \beta_1 x_{i1} + \cdots + \beta_p x_{ip} \geq M(1-\epsilon_i) \\ \epsilon_i \geq 0\,, \quad \sum_{i=1}^{n} \epsilon_i \leq C \end{cases}$$

C is a non-negative tuning parameter that gives information on the number of observations that can violate the margin. The variable ϵ_i are called *slack variables* and give information on the observation.

Ensemble Subspace Discriminant

In Ensemble Subspace Discriminant (ESD), a random selection of features in the subspaces and a majority voting (between the predictors) rule are used to build the ensemble of learners and to adopt the classification result. Ensemble predictors combine

results from many base learners, also referred to as ‘weak learners’, into one of higher performance, using methods such as bagging, subspace, boosting, etc.

The random subspace method is similar to bagging except that the features are randomly sampled, with replacement, for each learner. Informally, this causes individual learners to not over-focus on features that appear highly predictive/descriptive in the training set, but fail to be as predictive for points outside that set. For this reason, random subspaces are an attractive choice for high-dimensional problems where the number of features is much larger than the number of training points.

The random subspace method has been applied to different classifiers as discriminant classifiers.
